# Epidemiological Analysis and Susceptibility Profile of Pathogens Isolated in Blood Cultures From Hospital Angeles Lindavista in Mexico City

**DOI:** 10.7759/cureus.103199

**Published:** 2026-02-08

**Authors:** Mauricio Castillo-Salazar, Erika Perez Sanjulian-Krasovsky, María Citlali Martínez-Sánchez, José Luis Pinacho-Velázquez, Carlos Andrey Diosdado-Franco, Luis Gerardo Balcázar-Ochoa, Laura Gomez-Virgilio

**Affiliations:** 1 Laboratory and Blood Bank, Hospital Angeles Lindavista, Mexico City, MEX; 2 Medical Education, Hospital Angeles Lindavista, Mexico City, MEX; 3 Pediatrics, Hospital Angeles Lindavista, Mexico City, MEX; 4 General Management, Hospital Angeles Lindavista, Mexico City, MEX; 5 Physiology, Facultad de Medicina de la Universidad Nacional Autónoma de México, Mexico City, MEX; 6 Research and Teaching Coordination, Hospital Angeles Lindavista, Mexico City, MEX

**Keywords:** antimicrobial susceptibility, bloodstream infections, clinical predictors, empiric antibiotic selection, microbial resistance

## Abstract

Background

Bloodstream infections contribute to morbidity and mortality. A wide variety of microorganisms can be identified and recovered from these infections, and blood culture is the primary method for detecting them and determining their antimicrobial susceptibility. This helps clinicians confirm the efficacy of the initial empiric therapy or select an appropriate alternative based on the susceptibility of the isolated microorganism, thereby reducing the risks associated with antimicrobial misuse. We aimed to describe and analyze the bacteria isolated in blood culture samples, their antimicrobial resistance at Hospital Angeles Lindavista, and their association with demographic and clinical characteristics; to provide tools for the effective antimicrobial treatment of bacteremia; and to monitor the evolution of microbial resistance in this institution.

Methods

A retrospective observational study was conducted at Hospital Angeles Lindavista, including all patients with suspected sepsis, as determined by the physician’s judgement, who had at least one blood sample collected from January 2024 to December 2024. Variables examined included age, gender, clinical service, comorbidities, risk factors, diagnoses, detected pathogens, and antibiogram. The relationship between a specific bacterium and an affected organ system was analyzed using a chi-square test, followed by residual analysis. To explore potential associations between clinical variables, regression models were used. Finally, to verify the stability of the results, we performed a sensitivity analysis using only complete cases and compared these findings to those from the entire dataset. The statistical analysis was conducted using R version 4.4.1 (2024-06-14 ucrt) (R Foundation for Statistical Computing, Vienna, Austria) in RStudio (Posit Software, Boston, MA).

Results

We identified an adult population with a median age of 49 years and a predominance of men. The median hospital stay was eight days; hospitalization was the most common service, and the gastrointestinal system was most affected. Regarding risk factors, 31 patients (16%) had type 2 diabetes mellitus, and sepsis was the most frequent diagnosis (n = 18, 9%). *Escherichia coli* (*E. coli*) was the most common bacterium, isolated 17 times overall, with 11 producing extended-spectrum β-lactamases (ESBLs). These isolates showed high susceptibility to amikacin, cefotaxime, and tigecycline but exhibited marked resistance to all other antibiotics, including carbapenems. No statistically significant associations were found between any clinical predictor. It was suggested that ESBL-producing *E. coli* is linked to urinary tract infections; *Pseudomonas aeruginosa* appeared to be associated with multiorgan involvement, while *Staphylococcus aureus* was correlated with musculoskeletal infections.

Conclusions

The findings show that the epidemiology of bloodstream infections in our hospital remains steady, with consistent demographic patterns, a high rate of gram-negative organisms, and no clear clinical predictors of positive cultures. These results highlight the importance of microbiological surveillance, appropriate empiric antibiotic selection, and the careful interpretation of results, especially when potentially contaminating organisms are involved.

## Introduction

According to various studies, bloodstream infections are a significant cause of morbidity and mortality worldwide, and their incidence has increased in recent years, accompanied by epidemiological, etiological, and clinical changes [1,2]. This issue is associated with numerous adverse consequences for patients, including longer hospital stays, higher costs, and increased mortality rates [3]. A wide range of microorganisms can be identified and recovered from bloodstream infections, depending on several factors, including geographic location, infection-control practices at each hospital, antimicrobial use, and the patient population [4]. Currently, systemic infections constitute a significant complication in the hospital setting, especially in immunocompromised patients, with a high mortality rate [5].

The varied clinical presentation in patients with positive blood cultures can be explained by factors such as age, origin, the type of isolated microorganism, comorbidities, and antibiotic treatment [6]. Therefore, timely clinical recognition, the rapid detection of the microbiological agent, and appropriate antibiotic therapy are vital in the treatment of bloodstream infections [7]. Blood culture remains the first-line method for detecting microorganisms in the blood, as it detects, identifies, and determines the antimicrobial susceptibility of the causative [8].

Antimicrobial susceptibility testing enables clinicians to confirm the effectiveness of empiric therapy or select an alternative antimicrobial regimen based on the specific susceptibility profile of the isolated microorganism. This helps to reduce the consequences of antimicrobial misuse, primarily the development of resistant organisms and unnecessary increases in treatment costs [9]. Hence, guidelines for the empiric management of bloodstream infections are needed to ensure rapid and effective antibiotic regimens, which should be reviewed and updated regularly based on detailed local data on pathogen prevalence and antimicrobial resistance [10].

Blood culture plays a crucial diagnostic and prognostic role, as it is the only readily available laboratory method for detecting microorganisms in blood when infection is suspected, whether or not there is an obvious focus of infection [1-3]. This allows clinicians to confirm the selected empiric therapy or to choose an appropriate alternative antimicrobial treatment based on the susceptibility of the isolated microorganism or the patient’s characteristics [3]. This reduces the consequences of antimicrobial misuse, which ultimately leads to the selection of resistant microorganisms and significantly increases treatment costs [5,8]. Therefore, there is a need to quickly provide effective antibiotic regimens informed by detailed local data on pathogen prevalence and antimicrobial resistance.

In this context, we hypothesize that bloodstream infections at our institution exhibit a characteristic distribution of bacterial pathogens and antimicrobial resistance patterns that can be accurately identified through blood culture and susceptibility testing and that these locally defined epidemiological profiles provide clinically meaningful information capable of improving empiric antibiotic selection and reducing the risk of antimicrobial misuse. We aimed to describe and analyze the bacteria isolated from blood culture samples, their antimicrobial resistance at Hospital Angeles Lindavista, and their association with demographic and clinical characteristics.

## Materials and methods

Study design

From January 2024 to December 2024, a retrospective observational study was conducted at Hospital Angeles Lindavista to describe the pathogens isolated in blood cultures and their antibiotic susceptibility profile. The study included all patients with suspected sepsis, as determined by the physician, who had at least one blood sample collected within the specified time frame. It excluded patients whose blood samples were collected outside that period. Laboratory and clinical data were obtained from the Hospital Angeles Lindavista database electronic clinical history (ECH). Variables reviewed included age, gender, clinical service, comorbidities, risk factors, diagnoses, detected pathogens, and antibiogram.

To define a unique bloodstream infection episode, blood cultures drawn from the same patient on the same day were clustered, with only the first culture being considered for analysis; cultures drawn on different days were treated as separate episodes. To differentiate true infection from contamination, we applied microbiological criteria: an episode was classified as true infection if at least one blood culture grew a recognized pathogen (*Staphylococcus aureus* and *Escherichia coli *{*E. coli*}) at a concentration of >100,000 colony-forming units per milliliter (CFU/mL) or if the same organism was isolated from two or more separate cultures. Episodes that did not meet these criteria were reviewed for potential contamination. This was also applied to coagulase-negative staphylococci.

Laboratory testing

Blood samples were collected in appropriate blood culture bottles (aerobic, anaerobic, and/or pediatric) and incubated with the BactAlert system (bioMérieux, Marcy-l’Étoile, France) according to the manufacturer’s instructions. At least one set of aerobic and anaerobic blood culture bottles was taken from patients without an obvious source of pathogens and from those with an endovascular infection. Blood cultures were checked daily for seven days; if no microorganism was detected during this period, the result was reported as negative. When the result was positive, a smear was prepared and gram-stained, the isolate was plated on the appropriate medium, and the culture was incubated for 24-48 hours. Based on colony-forming unit (CFU) counts, identification and susceptibility testing were performed using the bioMérieux Vitek system. The results were reported both qualitatively (susceptible, intermediate, or resistant) and quantitatively (median inhibitory concentration). For the various microorganisms, the antimicrobial and technical safety specifications of the Clinical and Laboratory Standards Institute (CLSI) were used.

Data source

A retrospective database from Hospital Angeles Lindavista was collected during the specified period. Data on 661 blood cultures from 198 patients were used to create the database. The patient samples were assigned an automatically generated code, preserving their identities. Variable coding was standardized and grouped for statistical analysis.

Statistical analysis

The statistical analysis was performed using R version 4.4.1 (2024-06-14 ucrt) (R Foundation for Statistical Computing, Vienna, Austria) in RStudio (Posit Software, Boston, MA). Patient characteristics were reported as medians with interquartile ranges (IQR) for continuous variables and as absolute counts and percentages for categorical variables. For the analysis, patients were grouped by the clinical service where they were hospitalized, the affected organ system, and the site of blood culture collection (peripheral or central). The most common microorganisms identified in the blood cultures were described as absolute counts and percentages. Their susceptibility to clinically relevant antibiotics was expressed as the percentage of susceptible strains to each antibiotic. The association between a specific bacterium and an affected organ system, the latter of which was based on primary diagnosis and the clinician’s judgement, was evaluated using a chi-square test, followed by standardized residual (SR) analysis. To assess potential relationships between clinical variables, such as comorbidities, risk factors, and age, and outcomes, such as the length of hospitalization or culture results, linear generalized models were used. Linear regression was used for age, and logistic regressions were used for categorical variables (comorbidities, risk factors, and infections caused by specific microorganisms). Firth’s penalization was applied to the logistic regression models to address data separation and a small sample size. Finally, to test the stability of the findings, we conducted a sensitivity analysis using only complete cases, that is, patients with no missing data, and compared these results to those from the full dataset. A p-value of below 0.05 was considered statistically significant for all tests.

Ethical considerations

The study protocol was approved by the Research Ethics Committee of Hospital Angeles Pedregal (approval number: HAP 2785). All procedures were conducted in accordance with the ethical standards outlined in the Declaration of Helsinki. The committee granted a waiver of informed consent for this retrospective study because of its minimal-risk design. Confidentiality and data protection were maintained throughout the study.

## Results

Demographic and clinical variables

A total of 198 patients were included in our analysis. Age, gender, the length of hospital stay, and the hospital service responsible are summarized in Table 1.

**Table 1:** Description of the demographic variables, length of hospital stay, and associated hospital service. SD, standard deviation; IQR, interquartile range

Variables	Frequency
Age, mean (SD)	49 (34-66)
Gender	
Female, n (%)	88 (44%)
Male, n (%)	110 (56%)
Length of hospital stay (days), median (IQR)	8 (5-16)
Hospital service	
Hospitalization, n (%)	65 (33%)
Intensive care unit (ICU), n (%)	52 (26%)
Intermediate care unit, n (%)	30 (15%)
Emergency department, n (%)	7 (3.5%)

Missing data were documented for several variables: hospital service (22%, n = 44), affected system (19%, n = 37), risk factors (17%, n = 34), diagnosis (17%, n = 34), and sample classification (missing for nine, 22, and 26 patients in the single-sample, multiple-sample-same-day, and multiple-sample-different-day categories, respectively). All subsequent analyses are based on available data.

The analysis identified an adult population with a median age of 49 years (IQR: 34-66) and a predominance of men. Additionally, the median length of hospital stay was eight days (IQR: 5-16). Hospitalization was the most common service, primarily involving internal medicine, urology, and cardiology, followed by the intensive care unit (ICU) and the intermediate care unit.

The median age of the participants was 49 years, with quartiles at 34 and 66 years. Regarding gender, 88 patients were women (44%). The median hospital stay was eight days, with the five-day and 16-day quartiles being one and three, respectively; 44 observations were missing. The most frequently recorded hospital services were hospitalization, internal medicine, urology, and cardiology. The ICU accounted for 52 patients (26%), the intermediate care unit for 30 patients (15%), and the emergency department for seven patients (3.5%). Hospital service data were missing for 44 patients (22%).

The frequency of patients with specific organ systems affected, based on clinical history and risk factors, is shown in Table 2.

**Table 2:** Description of affected organ systems and risk factors of the study population. The table shows the percentage of patients with a determined affected organ system based on the major diagnosis. It also depicts the principal risk factors reported in the clinical archive.

Variables	Frequency
Affected systems	
Gastrointestinal, n (%)	33 (17)
Respiratory, n (%)	32 (16)
Neurologic, n (%)	23 (12)
Urinary, n (%)	19 (9.6)
Multiorganic, n (%)	17 (8.6)
Cardiovascular, n (%)	14 (7.1)
Musculoskeletal, n (%)	13 (6.6)
Endocrinologic, n (%)	5 (2.5)
Reproductive, n (%)	4 (2)
Dermatologic, n (%)	1 (0.5)
Risk factors	
None, n (%)	103 (52)
Type 2 diabetes mellitus, n (%)	31 (16)
Hypertension, n (%)	22 (11)
Smoking, n (%)	4 (2.0)
Obesity, n (%)	3 (1.5)
Cancer, n (%)	1 (0.5)
Hypothyroidism, n (%)	1 (0.5)
HIV, n (%)	1 (0.5)

The gastrointestinal system was the most affected, with 33 (17%) patients, followed by the respiratory system, involved in 32 (16%). The neurologic system was affected in 23 (12%) patients, the urinary system in 19 (9.6%), and the cardiovascular system in 14 (7.1%). Notably, 17 (8.6%) patients experienced multiorgan involvement. Missing data for affected systems were reported in 37 (19%) patients. Regarding risk factors, 103 (52%) patients reported no risk factors, while 31 (16%) had type 2 diabetes mellitus, and 22 (11%) had hypertension. Other less common risk factors included cancer, hypothyroidism, obesity, and HIV infection (each affecting one patient, 0.5%).

**Figure 1:**
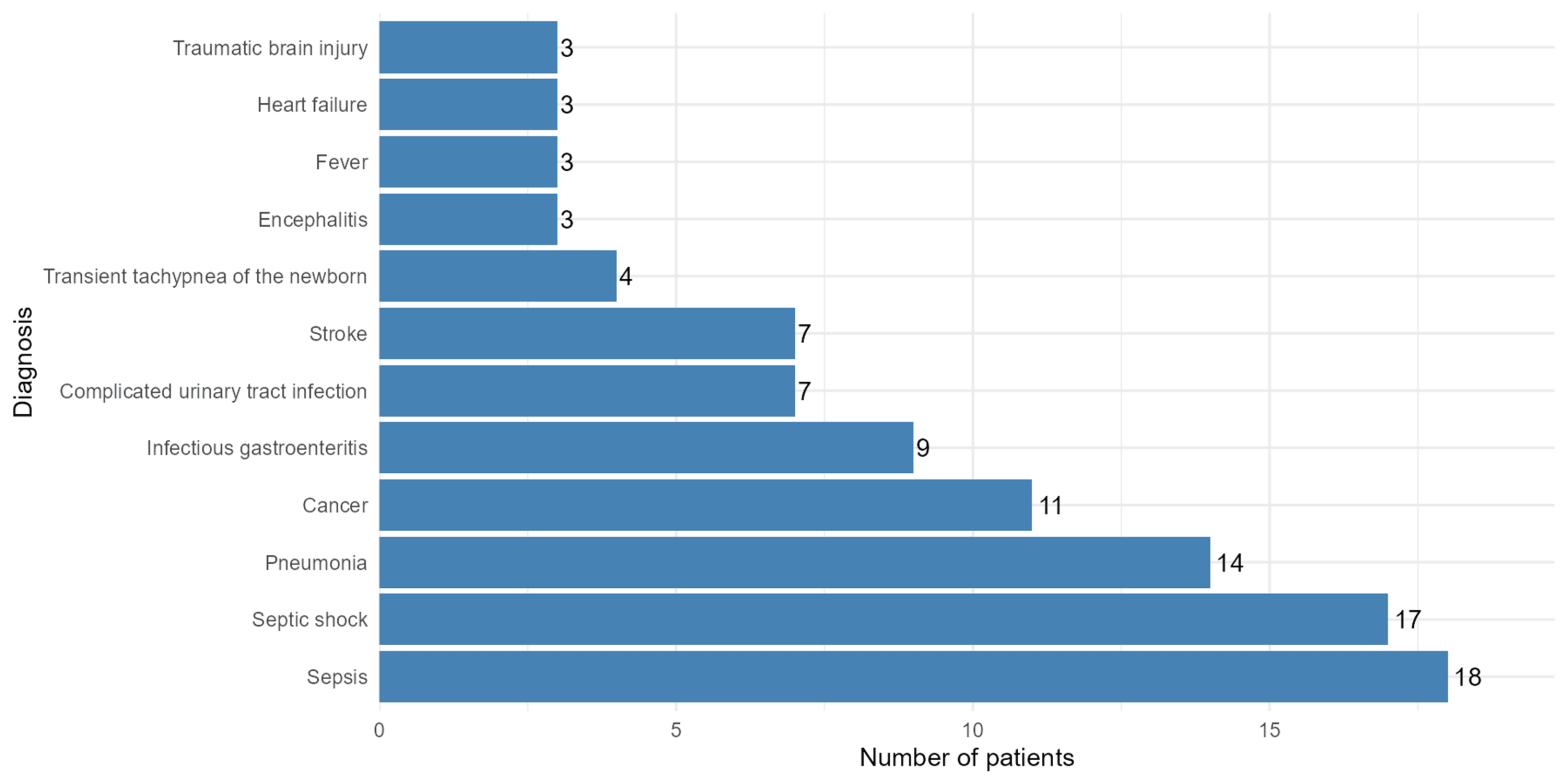
Summary of the most frequent diagnoses among patients who had blood cultures performed. Sepsis, septic shock, and pneumonia were the most frequent conditions observed, highlighting sepsis-related conditions as the leading causes of hospitalization.

Figure 1 lists the most common diagnoses among the participants. Sepsis was the most frequent diagnosis (n = 18, 9%), followed by septic shock (n = 17, 8.5%), pneumonia (n = 14, 7%), cancer (n = 11, 5.5%), infectious gastroenteritis (n = 9, 4.5%), complicated urinary tract infection (n = 7, 3.5%), stroke (n = 7, 3.5%), transient tachypnea of the newborn (n = 4, 2%), and encephalitis, fever, heart failure, and traumatic brain injury (each with n = 3, 1.5%).

Microbiological characteristics

A total of 661 blood cultures were performed on 198 patients. For most patients (55%), between one and two samples were drawn, whereas a smaller proportion had several samples collected on different days. Specifically, 64 patients had two samples, 45 had one sample, and 17 had three. Only a few patients (<5%) had more than six blood cultures during their hospitalization. The results were classified as single samples, multiple samples taken on the same day, or multiple samples collected on different days for the same patient. These data were further categorized by whether the blood sample was obtained from a peripheral or central catheter and by the blood culture result (Table 3).

**Table 3:** Distribution of blood samples based on blood sample source (central versus peripheral catheter) and sampling strategy (single sample, multiple samples on the same day, or samples obtained on different days). Most samples were collected from peripheral catheters. Blood cultures obtained on multiple dates showed the highest positivity rate.

Sample type	Single sample (n = 45)	Multiple samples, same day (n = 234)	Multiple dates (n = 382)
Central	12 (27%)	76 (32%)	152 (40%)
Peripheral	24 (53%)	136 (58%)	204 (53%)
Unknown	9 (20%)	22 (9.9%)	26 (7%)
Results			
Negative	40 (89%)	208 (89%)	316 (83%)
Positive	5 (11%)	26 (11%)	66 (17%)

Results showed there were 45 single samples, 234 multiple samples collected on the same day, and 382 multiple samples collected over multiple days. Central catheter samples accounted for 12 (27%) of the single samples, 76 (32%) of the multiple samples collected in a single day, and 152 (40%) of the multiple samples collected through several days. Correspondingly, a total of five (11%) single samples, 26 (11%) multiple samples collected in a single day, and 66 (17%) multiple samples collected through several days were positive.

**Figure 2:**
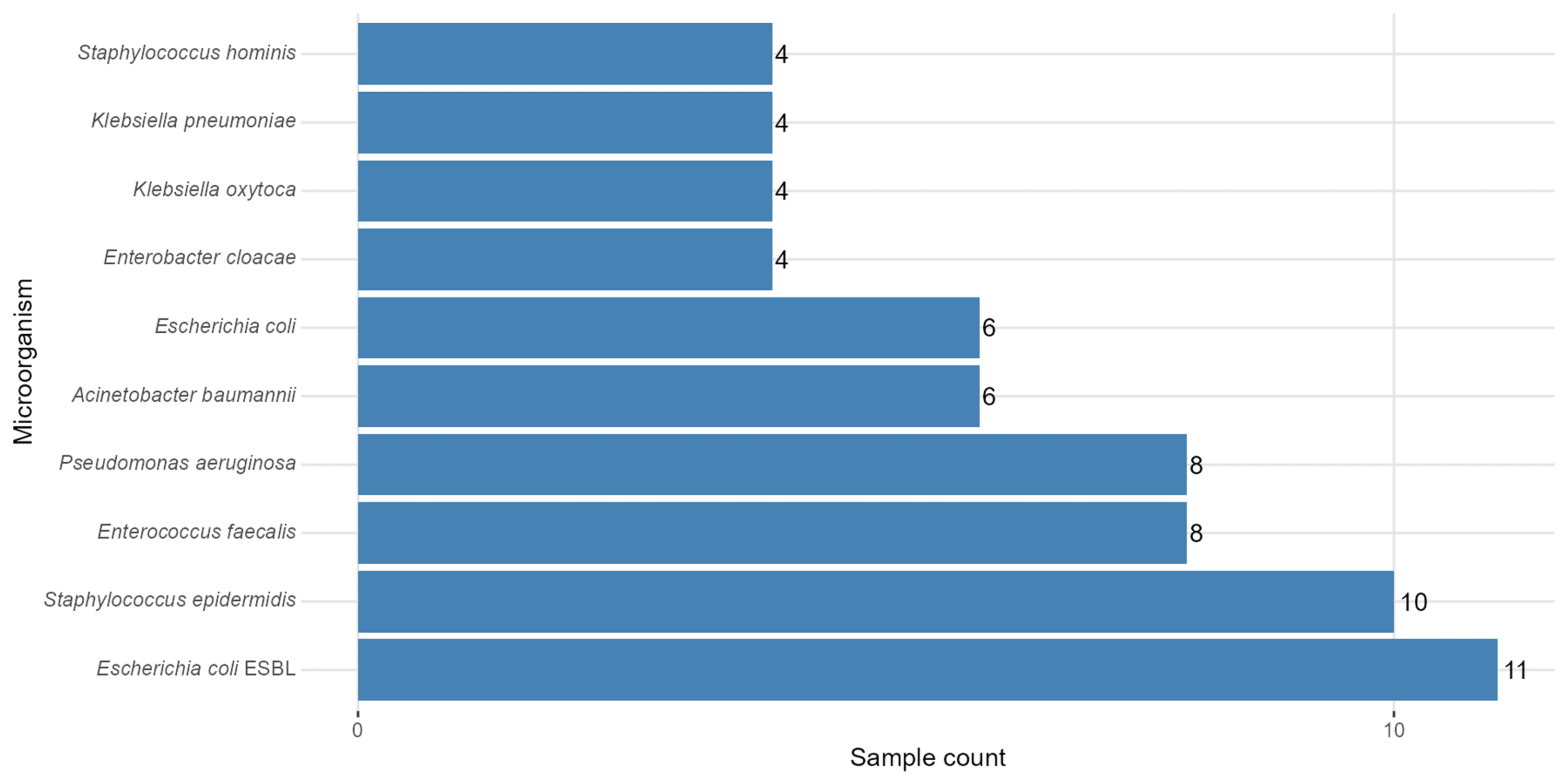
Distribution of microorganisms isolated from positive blood cultures. The most common pathogens were *Escherichia coli* (including ESBL-producing strains) and *Staphylococcus epidermidis*. ESBL: extended-spectrum β-lactamase

Figure 2 shows the number of blood culture isolates for the most common bacterial species. *Escherichia coli* was the most common bacterium, isolated 17 times in total, of which 11 were producers of extended-spectrum β-lactamases (ESBLs). The second most frequently isolated was *Staphylococcus epidermidis*, isolated 10 times, followed by *Enterococcus faecalis* and *Pseudomonas aeruginosa*, each isolated eight times. *Acinetobacter baumannii* was isolated six times, and *Enterobacter cloacae*, *Klebsiella oxytoca*, *Klebsiella pneumoniae *(*K. pneumoniae*), and *Staphylococcus hominis* were all isolated four times each.

**Figure 3:**
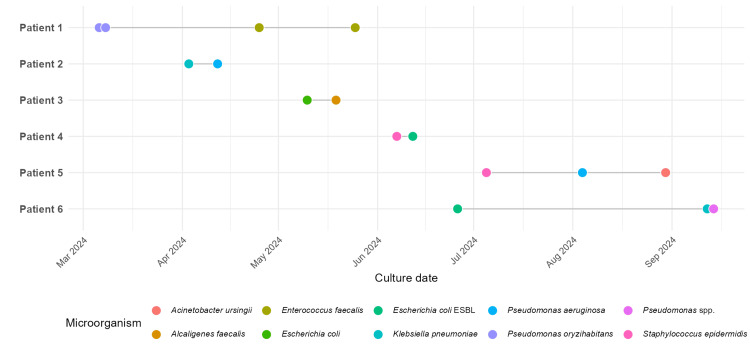
Timeline of positive blood cultures for six patients, showing the isolation of different microorganisms on separate dates. This pattern is consistent with possible superinfection or sequential bloodstream infection episodes, though the small sample size limits definitive conclusions. ESBL: extended-spectrum β-lactamase

To identify potential superinfection patterns, we selected patients with multiple blood cultures drawn on different days who showed a change in the isolated pathogen between those days. Six patients met this criterion (Figure 3).

The putative superinfecting microorganisms identified in serial blood cultures included *Enterococcus faecalis*, *Pseudomonas aeruginosa*, Alcaligenes faecalis, ESBL-producing *E. coli*, and *Acinetobacter ursingii*. These patients generally had a prolonged hospital stay.

**Figure 4:**
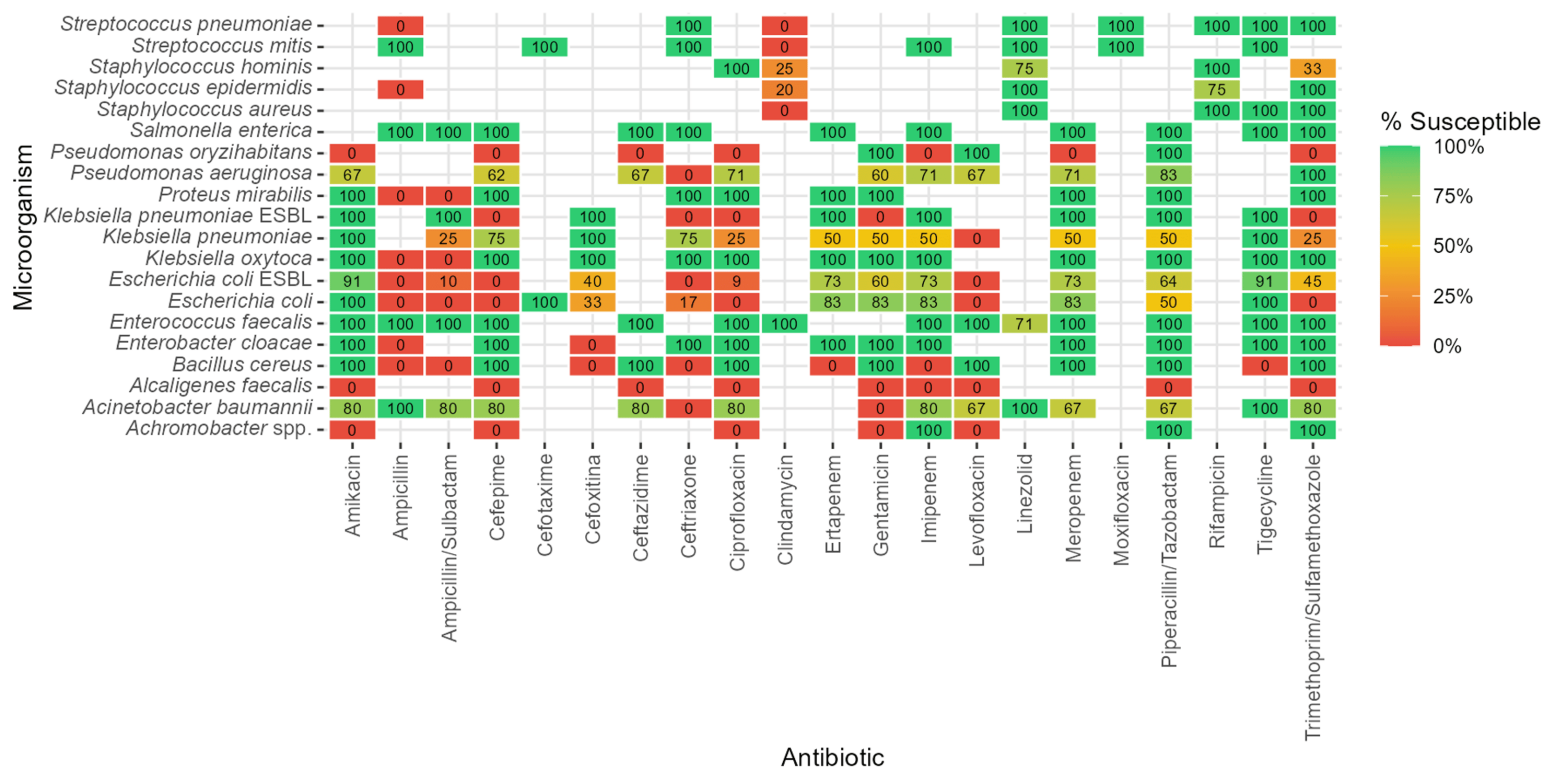
Antibiotic susceptibility profile of pathogens isolated from positive blood cultures. Susceptibility values indicate the percentage of isolates classified as susceptible for each organism-antibiotic combination. ESBL: extended-spectrum β-lactamase

Figure 4 shows a heatmap summarizing bacterial susceptibility to clinically relevant antibiotics.

Each cell indicates the percentage of susceptible isolates, calculated as the proportion of susceptible strains relative to the total number of isolates tested for a given antibiotic. Notably, *Pseudomonas aeruginosa* exhibited a moderate degree of resistance to most antibiotics tested but remained fully susceptible to trimethoprim/sulfamethoxazole (TMP/SMX). ESBL-producing *E. coli* showed high susceptibility to amikacin and tigecycline but marked resistance to all other antibiotics, including carbapenems. In contrast, non-ESBL-producing *E. coli* displayed greater susceptibility to cefotaxime, carbapenems, and TMP/SMX while maintaining susceptibility to amikacin. *Acinetobacter baumannii* demonstrated high susceptibility to ampicillin, linezolid, and tigecycline and moderate-to-high susceptibility to amikacin, cephalosporins, imipenem, and TMP/SMX. *Staphylococcus aureus* was entirely susceptible to linezolid, rifampicin, tigecycline, and TMP/SMX, similar to *Staphylococcus epidermidis*, which was also susceptible to linezolid and TMP/SMX. In contrast, *Staphylococcus hominis* was susceptible to ciprofloxacin and rifampicin but resistant to TMP/SMX. Finally, *Enterococcus faecalis* was highly susceptible to all antibiotics tested.

Table 4 and Table 5 present the results of linear and logistic regression models, respectively, which explored the associations between clinical variables, the length of hospital stay, and culture results.

**Table 4:** Linear regression model evaluating the association between clinical variables and the length of hospital stay. No variables were significantly associated with the outcome. The significant intercept reflects the baseline outcome value and is not clinically meaningful. ***Highly significant

Variables	Estimate	Standard error	T value	P-value
Intercept	13.10	2.38	5.51	<0.001***
Age	-0.019	0.050	-0.38	0.702
Diabetes	2.85	2.69	1.06	0.291
Hypertension	-1.30	3.08	-0.42	0.673
Other risk factor	3.05	3.75	0.81	0.418

**Table 5:** Logistic regression model evaluating the association between clinical variables and the result of blood culture. ***Highly significant

Variables	P-value	Odds ratio
Intercept	<0.001***	0.13
Age	0.641	1.00
Diabetes	0.786	0.86
Hypertension	0.087	2.62
Other risk factors	0.808	0.82

No statistically significant associations were found between any clinical predictor and the length of hospital stay in the linear regression model (Table 4). Although the model intercept was statistically significant, this indicates only that the baseline value of the outcome differed from zero and does not imply a clinically meaningful association.

In the logistic regression model (Table 5), none of the clinical predictors were statistically significant predictors of a positive blood culture result. Although hypertension showed a borderline p-value (p = 0.087), this did not reach statistical significance. The significant intercept reflects only the baseline odds of a positive culture when all predictors are equal to zero and is not clinically relevant.

**Figure 5:**
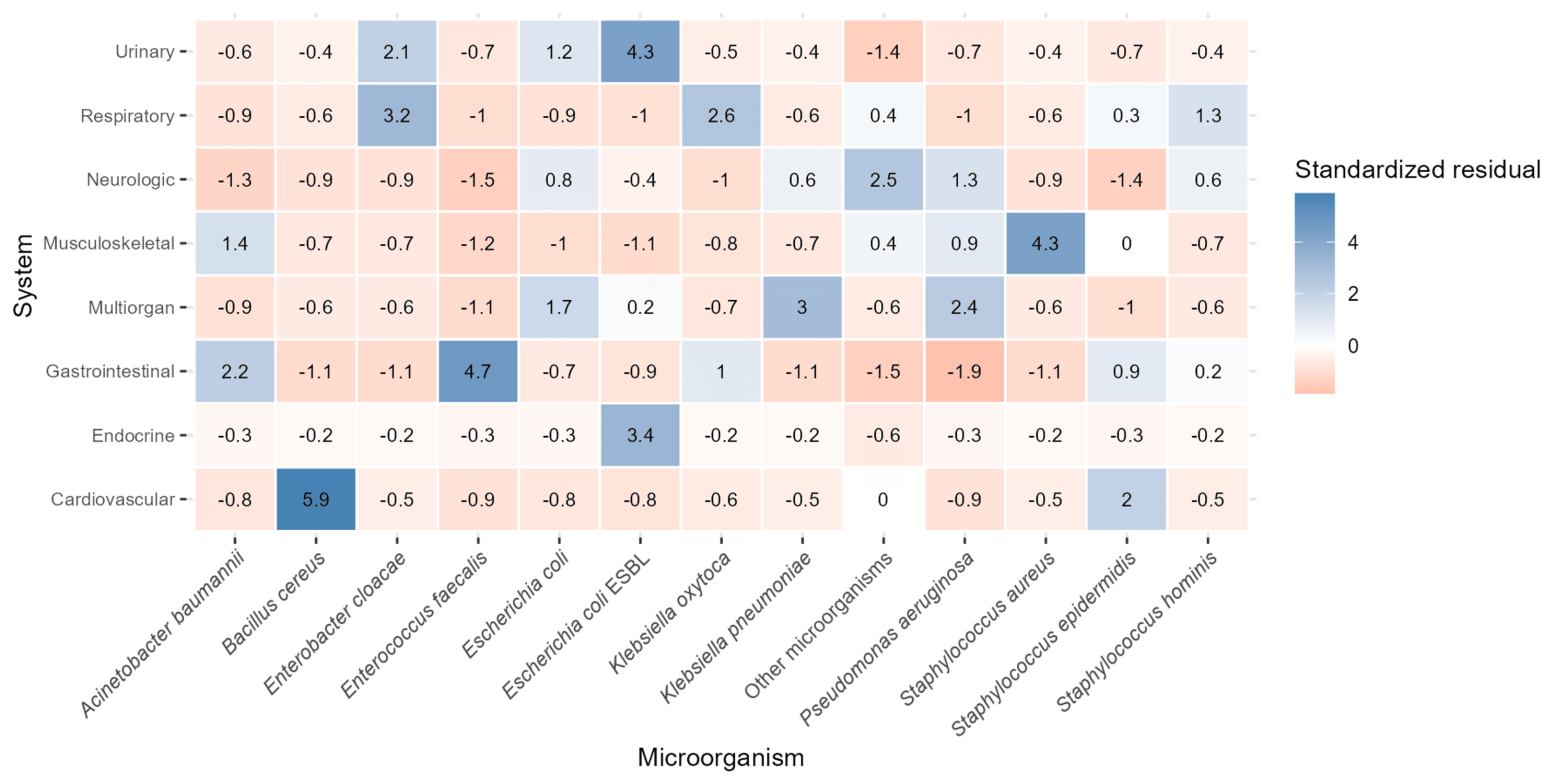
Heatmap of standardized residuals from the chi-square test assessing the association between the affected organ system and specific microorganisms isolated from blood cultures. Positive residuals (blue) indicate organism-system combinations observed more frequently than expected, whereas negative residuals (red) indicate fewer observed cases than expected.

Associations between the patients’ affected organ systems and specific bacterial species were investigated through a chi-square test. The results indicated differences in bacterial strains and in the particular organ system affected (p ≤ 0.05); therefore, we sought to characterize these differences using a standardized residual test (Figure 5).

Results are interpreted as follows: a standardized residual of >2 indicates a positive deviation from expected frequency, whereas a standardized residual of <-2 indicates a negative deviation. For instance, ESBL *E. coli* showed a standardized residual (SR) of 4.3 in the urinary system, indicating that it occurred more frequently than expected in urinary cases. *Pseudomonas aeruginosa* was overrepresented in multiorgan involvement (SR = 2.4), while *Staphylococcus aureus* was overrepresented in musculoskeletal affection (4.3). Other microorganisms, including *Acinetobacter ursingii*, ESBL *Klebsiella pneumoniae*, and *Streptococcus pneumoniae*, were overrepresented in neurologic affection (SR = 2.5). These residuals represent statistical associations in our sample rather than established clinical relationships.

Sensitivity analysis

To assess the robustness of the results and rule out potential biases introduced by sample selection or missing data, three complementary sensitivity analyses were performed.

First, an analysis was conducted comparing the original cohort (n = 198) to the sub-cohort of complete cases (n = 154), excluding 21.8% of patients with missing data for any clinical variable to evaluate the impact of the missing data (incomplete cases). Despite the reduction in sample size, the main demographic and clinical characteristics remained stable. The median age (49 versus 48 years) and the median length of hospital stay (eight days in both groups) showed no variation. Likewise, the absolute prevalence of major comorbidities such as diabetes mellitus and hypertension remained almost entirely unchanged in the complete case group.

Additionally, the microbiological profiles of patients included in the multivariate model (n = 83 positive) were compared to those of patients excluded due to incomplete data (n = 17 positive). Fisher’s exact test showed no significant differences in the distribution of the main pathogens (p = 0.2), indicating that patients with incomplete records did not have a different infectious profile. These findings suggest that the data loss was random and did not introduce systematic bias into the study population.

Second, to validate the methodological decision to analyze only the first positive blood culture per patient (single-patient analysis) using multivariate models, the demographic characteristics of this cohort were compared to those of all recorded blood culture events. No significant deviations were observed, confirming that selecting the first episode is representative of the general hospital population and does not overrepresent patients with prolonged stays.

Finally, the consistency of pathogen frequency was assessed by excluding microorganisms commonly associated with skin contamination (e.g., coagulase-negative staphylococci and *Bacillus* spp.). Although the absolute number of isolates decreased, the predominance of gram-negative bacilli (*Escherichia coli*, *Pseudomonas aeruginosa*, and *Klebsiella pneumoniae*) remained unchanged, underscoring their continued clinical priority in this hospital.

Notably, a detailed review of the excluded isolates confirmed that several coagulase-negative *Staphylococcus* strains had colony counts exceeding 100,000 CFU, a threshold consistent with actual bloodstream infection rather than contamination. This finding indicates that even under a conservative scenario, excluding pathogens did not alter the main epidemiological pattern.

## Discussion

This single-center retrospective study provides an epidemiological overview of bloodstream infections in a tertiary-care private hospital in Mexico, integrating clinical and microbiological variables and multivariate generalized linear models. This approach aims to generate insights into clinical decision-making regarding sepsis and antibiotic use in similar settings.

Regarding sampling strategy, we observed the highest positivity rate in blood cultures drawn over multiple days (17%), compared to single samples (11%) and multiple samples collected on the same day (11%). This descriptive pattern merits further investigation with comparative statistical tests.

Our results showed that gram-negative bacilli are the predominant cause of bloodstream infections in our hospital. Bacteria such as *Escherichia coli*, *Enterococcus faecalis*, *Pseudomonas aeruginosa*, and *Klebsiella pneumoniae* were among the most common isolates, consistent with global reports of bloodstream pathogens [11].

It is worth noting that extended-spectrum β-lactamase (ESBL)-producing strains, such as ESBL-producing *E. coli* and *K. pneumoniae*, were frequently isolated from positive blood cultures. This finding underscores the formidable problem of antibiotic resistance and prompts us to develop health policies and internal consensus to improve antibiotic use in hospitals.

Although gram-negative bacteria dominated the frequency analysis, coagulase-negative staphylococci were also commonly detected. Strains such as *Staphylococcus epidermidis* and *Staphylococcus hominis* were among the 10 most frequently identified isolates. These strains are commonly associated with sample contamination because they are abundant in the normal skin microbiota. Nonetheless, our review of the laboratory results revealed that these isolates exceeded 100,000 CFU, supporting their classification as bloodstream infections and not contamination. These results highlight the importance of conducting a detailed review of laboratory results to establish a well-founded treatment and avoid a pattern-based prescription (e.g., coagulase-negative *Staphylococcus* = contamination).

In our local setting, we observed a distinctive susceptibility pattern for *Pseudomonas aeruginosa*: low susceptibility to aminoglycosides, quinolones, and carbapenems but unexpectedly high susceptibility to trimethoprim/sulfamethoxazole (TMP/SMX). This local finding contrasts with typical resistance patterns and warrants confirmation in larger, multicenter studies before considering therapeutic implications [12]. Notably, other common gram-negative pathogens such as *E. coli* and *Klebsiella pneumoniae* showed expected resistance to TMP/SMX in our cohort [13].

The low levofloxacin susceptibility observed across multiple bacterial species at our center may reflect local resistance patterns that could limit its empiric utility.

It is suggested that the improper prescription of antibiotics is associated with their empiric use, based on faulty syndromic diagnoses [14]. In our results, the organ system-microorganism association identified through standardized residual analysis provided insights into this concern. Certain pathogens appeared disproportionately associated with specific systems, such as *Klebsiella pneumoniae* with cardiovascular presentations and ESBL-producing *E. coli* with gastrointestinal and urinary infections, suggesting potential patterns in infection pathogenesis or hospital-specific epidemiology. While these findings should be interpreted cautiously, they may help guide empiric therapy when the clinical syndrome is clearly defined, but culture results remain pending.

Despite the thorough examination of demographic and clinical variables, linear and logistic regression analyses found no statistically significant associations with culture positivity or the length of hospital stay. These results are consistent with previous reports suggesting that although common comorbidities such as type 2 diabetes or hypertension are relevant to patients’ clinical course, they are not, by themselves, strong predictors of bloodstream infections [15,16].

Moreover, the robustness of our results is confirmed by our multiple sensitivity analyses. The comparison between the original and complete-case-only populations revealed that patients with missing data did not introduce systematic bias. The sensitivity analysis also revealed that the demographic and clinical variables remained stable and that the microbiological profile of patients with missing data did not differ from that of the rest of the population. The decision to analyze only the first positive blood culture per patient was also validated, as this group was representative of the broader population and avoided overcounting individuals with prolonged or complicated hospitalizations.

We acknowledge several limitations. This was a retrospective, single-center study that reflects the epidemiological and microbiological features of a specific tertiary-care private hospital and may not be applicable to other institutions with different patient populations, referral patterns, or antimicrobial stewardship practices. Data from electronic clinical records highlight the potential for incomplete or imprecise documentation, which may affect the accuracy of clinical variables and diagnostic classifications. The decision to analyze only the first positive blood culture per patient avoided the overrepresentation of individuals with long hospital stays but may have limited the detection of polymicrobial infections or changes in pathogen dynamics over time. Automated susceptibility testing and CLSI breakpoints provide reliable guidance but may not detect emerging or rare resistance mechanisms, which could influence the interpretation of local susceptibility patterns. Additionally, while sensitivity analyses indicated that missing data did not introduce systematic bias, incomplete records naturally reduce statistical power and may obscure subtle associations. Finally, the one-year study period offers a helpful snapshot of local epidemiology but may not capture seasonal variations or long-term trends in antimicrobial resistance. Continued surveillance across multiple centers and over longer periods will be essential to validate these findings and support the development of durable, evidence-based antibiotic guidelines.

## Conclusions

Taken together, these findings highlight the importance of microbiological surveillance, appropriate empiric antibiotic selection, and the cautious interpretation of results when potentially contaminating organisms are present. These results provide a basis for developing a local evidence-based guideline for antibiotic therapy in blood infections, with the potential to improve bacterial resistance and patient outcomes. The periodic epidemiological characterization of blood cultures will have to be performed in our hospital to assess the evolution of bacterial susceptibility to antibiotics after the implementation of such guidelines.
